# The Association Between Cardiometabolic Risk Factors and Frailty in Older Adults: A Systematic Review

**DOI:** 10.1093/geroni/igac032

**Published:** 2022-05-25

**Authors:** Shamatree Shakya, Rashmita Bajracharya, Leila Ledbetter, Michael P Cary

**Affiliations:** School of Nursing, Duke University, Durham, North Carolina, USA; School of Medicine, University of Maryland, Baltimore, Maryland, USA; School of Medicine, Medical Center Library and Archives, Duke University, Durham, North Carolina, USA; School of Nursing, Duke University, Durham, North Carolina, USA

**Keywords:** Cardiometabolic risk factors, Frailty, Inflammation, Older adults

## Abstract

**Background and Objectives:**

Enhanced management and prevention of frailty depend on our understanding of the association between potentially modifiable risk factors and frailty. However, the associations between potentially modifiable cardiometabolic risk factors and frailty are not clear. The purpose of this review was to appraise and synthesize the current evidence examining the associations between the cardiometabolic risk factors and frailty.

**Research Design and Methods:**

Multiple databases, including MEDLINE (via PubMed), Embase (via Elsevier), and Web of Science (via Clarivate), were searched extensively. Studies that examined cardiometabolic risk factors and frailty as main predictors and outcome of interest, respectively, among older adults (≥60 years) were included. The Joanna Briggs Institute critical appraisal tools were used to evaluate the quality of studies. PRISMA (2020) guided this review, and findings were synthesized without meta-analysis. This systematic review was registered in PROSPERO (CRD42021252565).

**Results:**

Twelve studies met the eligibility criteria and were included in the review. Abdominal obesity, hyperglycemia, and multiple co-occurring cardiometabolic risk factors were associated with the increased likelihood of frailty in older adults. There was inconsistency across the studies regarding the associations between dyslipidemia, elevated blood pressure, and frailty.

**Discussion and Implications:**

Understanding the association between cardiometabolic risk factors and frailty can have translational benefits in developing tailored interventions for the prevention and management of frailty. More studies are needed to validate predictive and clinically significant associations between single and specific combinations of co-occurring cardiometabolic risk factors and frailty.

**Translational Significance:** This review examined the patterns of associations between potentially modifiable cardiometabolic risk factors and frailty. This study highlights that abdominal obesity, hyperglycemia, and the co-occurrence of multiple cardiometabolic risk factors are associated with an increased likelihood of frailty in older adults. A better understanding of the single and specific combination of cardiometabolic risk factors associated with frailty is needed to inform the precision-based health interventions for the prevention and management of frailty in older adults.

Frailty is a state of compromised homeostasis in multiple body systems, increasing the susceptibility to adverse health outcomes even in exposure to minor stressors (Clegg et al., 2013). The Fried phenotype model defines frailty as having at least three of these features: poor grip strength, gait speed, and physical activity; weight loss and fatigue. The manifestation of any one of these features is termed prefrailty―a precursor of frailty, and the absence of any features is a nonfrail state (Fried et al., 2001). Other commonly used frailty instruments, such as the Edmonton Frail Scale (Rolfson et al., 2006), the cumulative frailty index (Rockwood & Mitnitski, 2007), and the FRAIL scale (Thompson et al., 2020), also utilize the history of comorbidities, drug intake, and general health status to operationalize frailty. The prevalence of frailty is disproportionately higher among aging people (75 years and older), women, and certain race/ethnic subgroups (Bandeen-Roche et al., 2015; O’Caoimh et al., 2021). Specifically, the likelihood of frailty is greater among those living with chronic cardiometabolic diseases such as diabetes (García-Esquinas et al., 2015), cardiovascular diseases (Aguayo et al., 2019; Marinus et al., 2020), atrial fibrillation (Polidoro et al., 2013), heart failure (Denfeld et al., 2017), stroke (Palmer et al., 2019), and chronic kidney diseases (Chowdhury et al., 2017). Older adults with coexisting cardiometabolic diseases and frailty are less likely to tolerate advanced medical/surgical interventions such as anesthesia (Lin et al., 2018), cardioverter-defibrillator placement (Chen et al., 2019), surgery (Panayi et al., 2019), and chemotherapy (Runzer-Colmenares et al., 2020).

Although the exact mechanism underpinning the association between cardiometabolic diseases and frailty is unknown, several potential inflammatory mechanisms are underscored. Chronic inflammatory changes involved in cardiometabolic diseases are likely to increase the risk of frailty (Ferrucci & Fabbri, 2018). Chronic inflammatory changes and oxidative stress in cardiometabolic diseases (Amdur et al., 2016; Cesari et al., 2003) are marked by an increased level of inflammatory markers such as interleukin-6, C-reactive protein, and tumor necrosis factor-alpha (TNF-α) (Cesari et al., 2003; Marcos-Pérez et al., 2020). These inflammatory makers and sustained chronic inflammation are linked with frailty (Gale et al., 2013) and associated features such as declining muscle mass and strength (Bano et al., 2017). Some studies have also indicated that frailty is associated with an increased risk for cardiometabolic diseases, alluding to the likely bidirectional association between cardiometabolic diseases and frailty (Veronese, Cereda et al., 2017; Veronese, Sigeirsdottir et al., 2017). Thus, a greater understanding of the underlying association between potentially modifiable cardiometabolic risk factors and frailty can inform the strategies to delay and prevent frailty and adverse health outcomes.

Cardiometabolic risk factors include abdominal obesity (high waist circumference), insulin-resistant elevated blood glucose, dyslipidemia (low high-density lipoprotein [HDL]; elevated triglycerides and total cholesterol [TC]), and elevated blood pressure (Alberti et al., 2009). Cardiometabolic risk factors instigate a state of proinflammation and subacute systemic inflammation, altering homeostasis in multiple body systems (Noren et al., 2012), likely associated with frailty. Several observational studies have investigated the associations between cardiometabolic risk factors and frailty; however, no prior review has systematically evaluated and summarized the associations between a range of cardiometabolic risk factors and frailty. The intention of this review is not to delineate the mechanistic pathways between cardiometabolic risk factors and frailty. Instead, this review aimed to conduct a systematic review of the existing body of evidence to summarize the association between a range of potentially modifiable cardiometabolic risk factors and frailty in older adults. This review examined the patterns—similarities and differences in the unidirectional associations between potentially modifiable cardiometabolic risk factors and frailty. The findings of this systematic review can inform clinical interventions, clinical and public health practices to prevent and manage frailty.

## Method

Our initial search showed no prior studies or ongoing studies published or registered in PROSPERO and systematic reviews and meta-analyses indexed in PubMed on this topic. This systematic review protocol was registered at PROSPERO (registration no. CRD42021252565). This systematic review was carried out using the “Finding What Works in Health Care: Standards for Systematic Reviews” (Institute of Medicine [IOM], 2011). This systematic review was reported following the PRISMA 2020 statement: an updated guideline for reporting systematic reviews (Page et al., 2021).

### Data Sources and Search Strategy

The databases searched included MEDLINE (via PubMed), Embase (via Elsevier), and Web of Science (via Clarivate). The primary reviewer (S. Shakya) and a professional medical librarian (L. Ledbetter) outlined possible search terms defining study participants, concepts, and designs. Supplementary Tables 1 and 2 in Online Supplementary Material show the combinations of terms used to identify the potential studies. As shown in Supplementary Table 1, the professional librarian used a mix of keywords and subject headings representing cardiometabolic risk factors, cardiovascular risk factors, frailty, and older adult population, respectively. A search hedge to select for study types such as randomized control trials, prospective studies, and retrospective studies, as well as a database filter to remove publication types, such as editorials, letters, comments, and animal-only studies, was applied as was appropriate for each database. We did not limit the initial search by the date of publication or language of publication. Supplementary Table A shows the results of search term in the Web of Science (via Clarivate). The initial search was conducted by the librarian to find the articles from the date of inception to February 3, 2021. The librarian updated the search on November 19, 2021, to find any new articles. Thus, the search included articles from the date of inception to November 19, 2021. The search found a total of 10,550 citations. Complete reproducible search strategies, including date ranges and search filters, for all databases, are detailed in the Online Supplementary Material. We identified additional manuscripts by reviewing the reference list of prior systematic review (Amiri et al., 2020) and Google Scholar.

### Selection of Evidence Sources

**Figure 1.**
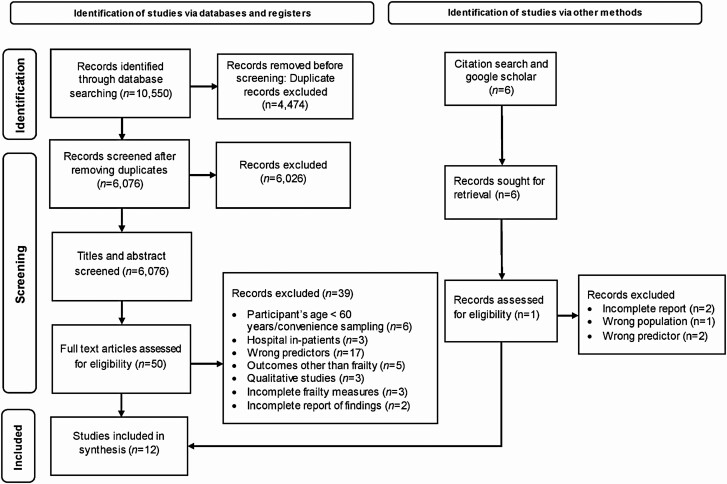
PRISMA diagram for search strategy and study selection process.

After the search, all identified studies were uploaded into Covidence (Veritas Health Innovation, Melbourne, Australia), a software system for managing systematic reviews, and 4,474 duplicates were removed by the software. A final set of 6,026 citations were left to be screened in the title/abstract phase, 50 articles were selected for the full-text review. From Google Scholar, six articles were selected for the full-text review. Of 56 articles deemed eligible for the full-text review, 12 studies met the inclusion criteria for data extraction, as shown in Figure 1 (Page et al., 2021). Two authors (S. Shakya and R. Bajracharya) independently carried out the selection process as presented in the flowchart as per PRISMA guidelines (Figure 1). The studies that met the eligibility criteria guided by PEOS (Population Exposure Outcome and Study types) guidelines were included in the final review.

#### Population

The cutoff points to classify the older adult population vary widely depending on countries’ eligibility criteria for social security and health care programs (Börscth-Supan & Coile, 2018); our review included those studies involving community-dwelling older adults (60 years and older) as the study participants, irrespective of country of origin.

#### Exposure

Cardiometabolic risk factors were the primary exposures of interest. Cardiometabolic risk factors include a group of potentially modifiable factors, including elevated waist circumference, blood glucose, blood pressure, triglycerides, TC, low-density lipoprotein (LDL), and reduced HDL (Alberti et al., 2009). Because there is some overlap between cardiometabolic risk factors and cardiovascular risk factors, cardiovascular risk factors were also used in the search strategy to capture cardiometabolic risk factors. Thus, this review includes studies involving a range of cardiometabolic risk factors.

#### Outcome

Only those studies that investigated frailty as the main study outcome were included in the review. Several valid tools exist, such as FRAIL scale, Edmonton Frail Scale, Tilburg, cumulative frailty index, and Fried phenotype model, using a combination of physical assessments and self-reported measures to operationalize frailty (Faller et al., 2019). Studies using the well-tested and valid instrument (Faller et al., 2019) for operationalizing frailty were included in this review, whereas those studies reporting partial or incomplete features (e.g., grip strength or gait speed only) to define frailty were excluded from the review.

#### Study types

All observational studies (including cross-sectional, prospective cohort, longitudinal, and case–control studies), experimental study designs (quasi-experimental and randomized clinical trials), and abstracts that provided quantitative statistical reports for describing the association between cardiometabolic risk factors and frailty were included in the review. Articles without empirical reporting were excluded (e.g., review articles, editorials, commentaries, conference proceedings, columns, or book chapters). In addition, articles were excluded if (a) cardiometabolic risk factors were examined as mediators or confounders and (b) there was a lack of explicit cardiometabolic risk factors. Every effort was made to obtain the full text of the articles, but where it could not be accessed, the article was excluded. For articles not published in English, due to restrictions in funding, we chose not to have these articles translated, and these articles were excluded at the full text-screening phase.

### Study Appraisal

The Joanna Briggs Institute (JBI) critical appraisal tools for cross-sectional and prospective cohort studies were used to appraise the methodological quality of retrieved studies. The two reviewers independently assessed the evaluation criteria in the JBI appraisal tool for cross-sectional/prospective cohort studies (Moola et al., 2020). The quality scores of individual articles were reflective of the sum of “yes” responses. The table showing study appraisal is available in the supplementary section (Supplementary Table 4).

### Data Extraction and Synthesis

The primary reviewer (S. Shakya) extracted data from each study using a standardized data-extraction form and recorded data in a standardized data-extraction file. The following data were extracted from each study: citation, publication year, country of origin, sample size, sampling techniques, study design, participants’ age, cardiometabolic risk factors, frailty assessment tools, covariates, and statistical associations indicating the strength and direction of association.

The included studies showed the variation in participants’ ages, origin, follow-ups, frailty measurements, and cardiometabolic risk factors and covariates, indicative of clinical heterogeneity. Similarly, the differences in sampling techniques and study designs across the included studies reflect methodological heterogeneity (Tables 1 and 2). Clinical and methodological heterogeneity can result in statistical heterogeneity, and aggregating statistical estimates in the presence of such heterogeneity can lead to inaccurate pooled estimate effects and misleading conclusions (Gagnier et al., 2012). Thus, for the present systematic review, we synthesized findings without meta-analysis. This approach included identifying patterns across the studies by critically analyzing similarities and differences across the study findings (Campbell et al., 2020).

## Results

### Study Characteristics

Twelve studies met the inclusion criteria and were included in the systematic review. As shown in Tables 1 and 2, the summary of study characteristics and findings is shown in the alphabetical order of the first author’s last name and the year of publication. Table 1 provides an overall summary of reviewed studies, including the origin of study, study design, and sampling techniques. The publication year ranged from 2007 to 2021 for reviewed studies (Anker et al., 2021; Barzilay et al., 2007). These studies were conducted in six different countries, including the United States (*n* = 5), China (*n* = 2), England (*n* = 1), Spain (*n* = 2), Switzerland (*n* = 1), and Taiwan (*n* = 1). Five studies used cross-sectional designs (Blaum et al., 2009; Crow et al., 2019; Lee et al., 2020; Liao et al., 2018; Song et al., 2020), and seven studies used longitudinal designs (Anker et al., 2021; Barzilay et al., 2007; Gale et al., 2014; Graciani et al., 2016; Kalyani et al., 2012; Pérez-Tasigchana et al., 2017; Zaslavsky et al., 2016). For the studies with a longitudinal design, the follow-up period ranged from 3.5 to 12 years (Anker et al., 2021; Pérez-Tasigchana et al., 2017). The number of study participants within a given study ranged from 346 to 6,320 (Kalyani et al., 2012; Liao et al., 2018). Most studies included a nationally representative population or population representing a state, county, or province. Stratified random sampling or random sampling was a frequently used sampling technique to recruit study participants, as shown in Table 1.

**Table 1.** Summary of study characteristics.

Study	Country	Predictors of Interest	Sample Size	Sampling Techniques	Study Design	Years of Follow-Up	Cohort Name	Participant’s Age
Anker et al. (2021)	Switzerland	Systolic and diastolic blood pressure	4,200	Random sampling	Longitudinal	12 years	Lausanne cohort Lc65+	65–70 years
Barzilay et al. (2007)	United States	Metabolic syndrome	2,826	Age- and sex-stratified random sampling	Prospective cohort Study	9 years	Cardiovascular Health Study	≥65 years
Blaum et al. (2009)	United States	Hyperglycemia	543	Age-stratified random sampling	Cross-sectional	―	Women’s Health and Aging Studies I and II	≥60 years
Crow et al. (2019)	United States	Abdominal obesity	4,984	Multistage, probability sampling design	Cross-sectional	―	National Health and Nutrition Survey (NHANES)	≥60 years
Gale et al. (2014)	England	Framingham cardiovascular risk	1,726	Stratified sampling	Longitudinal study	4 years	English Longitudinal study of Aging (ELSA)	≥60 years
Graciani et al. (2016)	Spain	Ideal cardiovascular risk	2,617	Stratified cluster	Prospective cohort study	3.5 years	Not available	≥60 years
Kalyani et al. (2012)	United States	Hyperglycemia	346	Age-stratified random sampling	Longitudinal design	8.6 ± 3.6 years	Women’s Health and Aging Study II	≥70 years
Lee et al. (2020)	Taiwan	Metabolic syndrome	1,006	Multiple proportional to size	Cross-sectional study	―	The Social Environment and Biomarkers of Aging Study (SEBAS)	≥65 years
Liao et al. (2018)	China	Abdominal obesity	6,320	Multistage sampling	Cross-sectional	―	Beijing Longitudinal Study on Aging	≥65 years
Pérez-Tasigchana et al. (2017)	Spain	Metabolic syndrome	1,499	Not available	Longitudinal prospective cohort study	3.5 years	Senior ENRICA	≥60 years and older
Song et al. (2020)	China	Abdominal obesity	995	Random multistage clustering sample	Cross-sectional	―	Not available	≥65 years
Zaslavsky et al. (2016)	United States	Hyperglycemia	1,848	Random sampling	Prospective cohort study	4.8 years	Adult Changes in Thought (ACT)	≥65 years

**Table 2.** Associations of Cardiometabolic Risk Factors and Frailty (*n* = 12)

Study	Cardiometabolic Risk Factors	Frailty Assessment Tool	Adjusted Variables	Statistical Analysis	Results
Anker et al. (2021)	Systolic and diastolic blood pressure	Fried frailty phenotype	Age, sex, education, BMI, hypertension, hypertension treatment, hypercholesterolemia, diabetes, CVD, smoking status, and polypharmacy	Multistate Markov model	Elevated systolic and diastolic blood pressures were not related to transition between different frailty statuses after adjusting for age, sex, education, BMI, hypertension, hypertension treatment, hypercholesterolemia, diabetes, CVD, smoking status, and polypharmacy.
Barzilay et al. (2007)	Metabolic syndrome and individual risk factor Blood pressure	Fried frailty phenotype	Age, sex, smoking status, education, income, marital status, BMI, depression, cognitive status, and clinical morbidities (diabetes mellitus, heart disease, stroke, and cancer)	Multivariate discrete time-proportional hazard models	In unadjusted and adjusted models after controlling for age, sex, smoking status, education, income, marital status, BMI, depression, cognitive status, and clinical morbidities, such as diabetes mellitus, heart disease, stroke, and cancer: • Cardiometabolic syndrome was associated with a new onset of frailty; • Elevated systolic blood pressure was not associated with the new onset of frailty. Insulin resistance-Homeostasis model assessment (IR-HOMA) was associated with a new onset of frailty.
Blaum et al. (2009)	Hyperglycemia	Fried frailty phenotype	Age, race, education, BMI, IL-6, and chronic diseases (osteoarthritis, coronary heart disease, stroke, diabetes mellitus, and COPD)	Multiple variable multinomial logistic regression	Hyperglycemia was significantly associated with a greater risk of frailty in the adjusted model controlling for BMI, IL-6, and chronic morbidities such as diabetes mellitus, osteoarthritis, coronary heart disease, stroke, and COPD.
Crow et al. (2019)	Abdominal obesity	Modified Fried frailty phenotype	Age, gender, race, ethnicity, marital status, education, BMI, and chronic diseases (diabetes, heart failure, CAD, and arthritis)	Multiple linear regression models and multiple logistic regression	Abdominal obesity was significantly associated with a greater risk of frailty in unadjusted and adjusted models controlling for age, gender, smoking, and education. It was not significantly associated with frailty after adjusting for age, gender, smoking, education, diabetes, heart failure, CAD, and arthritis.
Gale et al. (2014)	Framingham cardiovascular risk and individual risk factors: • total cholesterol • high-density lipoprotein Blood pressure	Modified Fried frailty phenotype	Cognitive function, household wealth, BMI, age, hypertension treatment, smoking, and diabetes	Multinominal logistic regression	In participants without CVDs, increased Framingham cardiovascular risk was significantly associated with greater risk of frailty and prefrailty in the adjusted model after controlling for age, antihypertensive treatment, smoking, and diabetes. In the adjusted model controlling for cognitive function, household wealth, BMI, age, hypertension treatment, smoking, and diabetes: • Elevated total cholesterol and reduced high-density lipoprotein (HDL) were significantly associated with greater risk of frailty; • Elevated systolic blood pressure was associated with greater risk of frailty.
Graciani et al. (2016)	Ideal cardiovascular risk - blood glucose - total cholesterol - BMI - blood pressure - smoking - physical activities - diet	Fried frailty phenotype	Model 1: sex, age, and education Model 2: sex, age, education, smoking, alcohol consumption, ADL, physical activity level, BMI, osteomuscular disease, chronic lung disease, and depression	Cox regression model	In the adjusted model controlling for sex, age, education, smoking, alcohol consumption, ADL, physical activity level, BMI, osteomuscular disease, chronic lung disease, and depression: • In participants without CVDs and diabetes, greater number of optimum cardiometabolic indicators were associated with lower risk of a new onset of frailty; • Optimum total cholesterol was not significantly associated with the new onset of frailty; • Optimum BMI was significantly associated with the new onset of frailty. Optimum untreated glucose level significantly associated with the lowered risk of new onset of frailty in the model adjusted for demographic factors; however, association was not statistically significant in the adjusted model controlling for demographic and chronic conditions. Optimum blood pressure was not associated with a new onset of frailty in the adjusted model controlling for sociodemographic characteristics, it was not associated with a new onset of frailty in the adjusted model controlling for chronic conditions, depression, and disabilities.
Kalyani et al. (2012)	Hyperglycemia	Modified Fried frailty phenotype	BMI, IL-6, and chronic morbidities (diabetes mellitus, osteoarthritis, COPD, CAD, and peripheral renal disease)	Cox regression model	Hyperglycemia was significantly associated with a new onset of frailty in the adjusted model controlling for BMI, IL-6, and chronic morbidities such as diabetes mellitus, osteoarthritis, COPD, CAD, and peripheral renal disease.
Lee et al. (2020)	Metabolic syndrome and individual risk factors - abdominal obesity - hyperglycemia - triglycerides - high-density lipoprotein - blood pressure	Frailty index of 35 items	Age, sex, education, smoking, and alcohol consumption status	Multiple logistic regression	In the adjusted model controlling for age, sex, education, smoking, and alcohol consumption status: • Metabolic syndrome, abdominal obesity, hyperglycemia, elevated triglycerides, and elevated blood pressure were significantly associated with greater risk of frailty; • Lowered HDL was not significantly associated with greater risk of frailty.
Liao et al. (2018)	Abdominal obesity	Rockwood’s 33-item frailty index	Sex, age, education, lifestyle factors―smoking, alcohol consumption, sleeping, physical activities, living alone, and number of chronic diseases (hypertension, diabetes mellitus, CVD, COPD, stroke, arthritis, tumor, dementia, heart failure, and renal failure)	Multiple logistic regression	Abdominal obesity and general obesity (excess BMI) were significantly associated with greater risk of frailty in unadjusted models. In the adjusted model controlling for sex, age, education, lifestyle factors―smoking, alcohol consumption, sleeping, physical activities, living alone, chronic diseases―hypertension, diabetes mellitus, CVD, COPD, stroke, arthritis, tumor, dementia, heart failure, and renal failure: • Abdominal obesity was significantly associated with greater risk of frailty; • General obesity (excess BMI) was not significantly associated with greater risk of frailty.
Pérez-Tasigchana et al. (2017)	Metabolic syndrome and individual risk factors - abdominal obesity - hyperglycemia - triglycerides - high-density lipoprotein - blood pressure	Fried frailty phenotype	Sex, age, education, diet, tobacco, alcohol consumption, physical activities, sedentary activities, total energy intake, asthma, chronic bronchitis, cancer, depression, musculoskeletal disease	Multiple logistic regression	In unadjusted and adjusted models controlling for sex, age, education, diet, tobacco, alcohol consumption, physical activities, sedentary activities, total energy intake, asthma, chronic bronchitis, cancer, depression, and musculoskeletal disease: • In participants without diabetes mellitus and CVD, cardiometabolic syndrome was significantly associated with the new onset frailty; • Abdominal obesity was significantly associated with the new onset of frailty; • Lowered HDL cholesterol did not significantly increase the risk of frailty; • Hyperglycemia and elevated blood pressure was not significantly associated with frailty. Elevated triglycerides level was significantly associated with the new onset of frailty in an unadjusted model. However, the association was not statistically significant in the adjusted model controlling for the aforementioned variables.
Song et al. (2020)	Abdominal obesity	Tilburg frailty indicator	Age, gender, marital status, educational level, and lifestyle factors: smoking, alcohol consumption, physical activity, diet, chronic diseases (cancer, CVD, COPD, diabetes, and others)	Multiple linear regression	Abdominal obesity was significantly associated with the greater risk of frailty in unadjusted and adjusted models controlling for age, gender, income, marital status, education, lifestyles, and chronic conditions.
Zaslavsky et al. (2016)	Hyperglycemia	Modified Fried frailty phenotype	Age, sex, race, education, depression level, smoking status, self-rated health, BMI, depression, cognitive functioning; chronic diseases (congestive heart failure, CAD, or COPD)	Cox regression model	Hyperglycemia was significantly associated with a new onset of frailty in two subgroups of participants with and without diabetes. In participants without diabetes, persistent higher glucose level (>100 mg/dl) was significantly associated with a new onset of frailty across 5 years. In participants with diabetes, the U-shaped association was observed between hyperglycemia, such that glucose lower than 160 mg/dl and higher than 180 mg/dl were significantly associated with the new onset of frailty across 5 years.

*Notes:* BMI = body mass index; CAD = coronary artery disease; CVD = cardiovascular disease; COPD = chronic obstructive pulmonary disease; IL-6 = interleukin-6; ADL = activities of daily living.

### Operationalization of Cardiometabolic Risk Factors and Frailty

Table 2 provides the summary of patterns―similarities and discrepancies in the associations between potentially modifiable cardiometabolic risk factors and frailty. Various instruments were used to operationalize frailty. Nine studies used the Fried or modified Fried frailty model (Anker et al., 2021; Barzilay et al., 2007; Blaum et al., 2009; Crow et al., 2019; Gale et al., 2014; Graciani et al., 2016; Kalyani et al., 2016; Pérez-Tasigchana et al., 2017; Zaslavsky et al., 2016), two studies used the cumulative frailty index (Lee et al., 2020; Liao et al., 2018), and one study used the Tilburg frailty indicator (Song et al., 2020) to operationalize frailty. The included studies examined the association between cardiometabolic risk factors, such as abdominal obesity (marked by elevated waist circumference), hyperglycemia (marked by elevated fasting glucose or glycated hemoglobin [HbA1C]), dyslipidemia (marked by elevated triglycerides, TC, or lowered HDL), and high blood pressure (marked by elevated systolic blood pressure), and frailty or development of frailty (new onset) in baseline nonfrail older adults. These studies examined the association of frailty with individual cardiometabolic risk factors or co-occurring cardiometabolic risk factors using measures such as cardiometabolic syndrome, ideal cardiovascular risk score, and Framingham cardiovascular risk score.

### Association Between Cardiometabolic Risk Factors and Frailty

#### Hyperglycemia and frailty

Hyperglycemia, indicated by elevated fasting blood glucose or glycated hemoglobin, was investigated as a risk factor of frailty in two cross-sectional studies (Blaum et al., 2009; Lee et al., 2020) and four prospective longitudinal studies (Graciani et al., 2016; Kalyani et al., 2012; Pérez-Tasigchana et al., 2017; Zaslavsky et al., 2016). Hyperglycemia was associated with an increased risk of frailty in two cross-sectional studies (Blaum et al., 2009; Lee et al., 2020) and three longitudinal studies (Graciani et al., 2016; Kalyani et al., 2012; Zaslavsky et al., 2016) after adjusting for sex, education, body mass index (BMI), interleukin-6, and chronic comorbidities such as diabetes mellitus, osteoarthritis, coronary heart disease, stroke, chronic pulmonary disease, and renal diseases, irrespective of participant’s diabetic status. Mainly, HbA1C above 6.5% was associated with the increased risk of frailty (Blaum et al., 2009; Kalyani et al., 2012). Only one study compared the association between hyperglycemia and frailty in subgroups of participants with and without diabetes mellitus, and hyperglycemia was associated with frailty in both subgroups (Zaslavsky et al., 2016). Persistent high fasting blood glucose (>110 mg/dl) was associated with the new onset of frailty in nondiabetic participants. In the diabetic participants, fasting blood glucose lower than 160 mg/dl and higher than 180 mg/dl were associated with the new onset of frailty (Zaslavsky et al., 2016). In two studies involving nondiabetic participants (Graciani et al., 2016; Pérez-Tasigchana et al., 2017), only one study showed that optimum blood glucose was associated with a reduced likelihood of developing frailty after adjusting for age, sex, and education (Graciani et al., 2016). Hyperglycemia was significantly associated with frailty features such as lower grip strength (Pérez-Tasigchana et al., 2017).

#### Abdominal obesity and frailty

Abdominal obesity, marked by sex-specified raised waist circumference, was examined as a risk factor of frailty in four cross-sectional studies (Crow et al., 2019; Lee et al., 2020; Liao et al., 2018; Song et al., 2020) and a longitudinal study (Pérez-Tasigchana et al., 2017). Abdominal obesity was consistently associated with the increased risk of frailty in all four cross-sectional (Crow et al., 2019; Lee et al., 2020; Liao et al., 2018; Song et al., 2020) and longitudinal (Pérez-Tasigchana et al., 2017) studies after adjusting for sociodemographic factors―age, sex, education, lifestyle factors―smoking, alcohol intake, physical activities, and chronic diseases such as diabetes mellitus, heart failure, coronary artery disease, chronic obstructive pulmonary disease, and cardiovascular diseases. In particular, abdominal obesity was associated with frailty features such as lowered grip strength and fatigue (Pérez-Tasigchana et al., 2017). Liao et al. (2018) compared the associations of abdominal obesity and general obesity (marked by greater BMI) with frailty. This study found that abdominal obesity was only associated with the risk of frailty when adjusted for sociodemographic and chronic conditions (Liao et al., 2018).

#### Dyslipidemia and frailty

Dyslipidemia was indicated using elevated triglycerides (Lee et al., 2020; Pérez-Tasigchana et al., 2017), TC (Gale et al., 2014; Graciani et al., 2016), and lowered HDL (Gale et al., 2014; Lee et al., 2020; Pérez-Tasigchana et al., 2017). The associations between dyslipidemia and frailty were examined in one cross-sectional (Lee et al., 2020) and three longitudinal studies (Gale et al., 2014; Graciani et al., 2016; Pérez-Tasigchana et al., 2017). There was inconsistency across the studies regarding the associations of dyslipidemia and frailty. Among one cross-sectional (Lee et al., 2020) and two longitudinal studies (Gale et al., 2014; Pérez-Tasigchana et al., 2017), only one longitudinal study indicated that lowered HDL was associated with the new onset of frailty after adjusting for age, BMI, smoking, diabetes, and cognitive status (Gale et al., 2014). Elevated triglycerides were shown to be associated with frailty in cross-sectional (Lee et al., 2020) and longitudinal studies (Pérez-Tasigchana et al., 2017) after adjusting for sociodemographic, lifestyles factors, and chronic morbidities. Elevated triglycerides were related to unintentional weight loss―a key feature of frailty (Pérez-Tasigchana et al., 2017). Among two longitudinal studies (Gale et al., 2014; Graciani et al., 2016), only one study showed the association between elevated TC level and new onset of frailty after adjusting for age, BMI, smoking, diabetes, and cognitive status (Gale et al., 2014).

#### Elevated blood pressure and frailty

Elevated blood pressure, marked by high systolic or diastolic blood pressure, was examined as a risk factor of frailty in one cross-sectional (Lee et al., 2020) and five longitudinal studies (Anker et al., 2021; Barzilay et al., 2007; Gale et al., 2014; Graciani et al., 2016; Pérez-Tasigchana et al., 2017). There were inconsistencies across the studies regarding the associations between elevated blood pressure and frailty after adjusting for sociodemographic and chronic diseases. Elevated systolic blood pressure was associated with the increased risk of frailty in one cross-sectional (Lee et al., 2020) and new onset of frailty in nonfrail older adults in one prospective study after adjusting for age, BMI, smoking, diabetes, hypertension treatment (Gale et al., 2014). However, optimum blood pressure was not associated with a new onset of frailty (Graciani et al., 2016). Similarly, elevated blood pressure did not influence transitions in nonfrailty, prefrailty, and frailty states over 12 years study period adjusting for age, sex, hypertension, hypertension treatment, hypercholesterolemia, diabetes, cardiovascular disease, and polypharmacy (Anker et al., 2021).

#### Co-occurring cardiometabolic risk factors and frailty

One cross-sectional (Lee et al., 2020) and four longitudinal studies (Barzilay et al., 2007; Gale et al., 2014; Graciani et al., 2016; Pérez-Tasigchana et al., 2017) examined the associations between multiple co-occurring cardiometabolic risk factors and frailty. The co-occurrence of multiple cardiometabolic risk factors indicated by cardiometabolic syndrome (Barzilay et al., 2007; Lee et al., 2020; Pérez-Tasigchana et al., 2017) and Framingham cardiovascular risk score (Gale et al., 2014) were associated with a greater likelihood of frailty and prefrailty adjusting for sociodemographic characteristics, smoking, alcohol consumption, and chronic diseases in one cross-sectional and four longitudinal studies. Even in participants without diabetes, cardiometabolic syndrome was associated with the new onset of frailty after adjusting for sociodemographic characteristics, lifestyles, depression, and chronic conditions (Pérez-Tasigchana et al., 2017). Similarly, in participants without diabetes and cardiovascular diseases, optimum cardiovascular health marked by ideal blood pressure, TC, blood glucose, and other protective factors such as nonsmoking status, normal body weight, and physically active lifestyle were associated with the reduced risk of frailty in older adults after adjusting for sociodemographic characteristics, lifestyles, BMI, and chronic conditions (Graciani et al., 2016).

## Discussion

Abdominal obesity, hyperglycemia, and the co-occurrence of multiple cardiometabolic risk factors were consistently associated with the increased risk of frailty across cross-sectional and longitudinal studies after adjusting for potential covariates: sociodemographic-age, sex, education, lifestyle factors―physical activity, smoking, tobacco use, alcohol intake, chronic conditions―diabetes mellitus, osteoarthritis, coronary heart disease, stroke, chronic pulmonary disease, and renal diseases. However, the associations between dyslipidemia, elevated blood pressure, and frailty were inconsistent across the included studies.

Our analysis extends the current literature by demonstrating that abdominal obesity, hyperglycemia, and co-occurrence of multiple cardiometabolic risk factors are associated with the increased likelihood of frailty or the development of frailty. Our findings align with the past systematic review that demonstrated a considerably greater risk of frailty in older adults with abdominal obesity (Yuan et al., 2021). Similarly, the association between hyperglycemia and frailty closely confirms that chronic uncontrolled diabetes contributes to frailty. In addition, an alternative indicator of hyperglycemia and insulin resistance, such as insulin resistance-homeostasis model assessment, could be used to examine the risk of frailty (Barzilay et al., 2007). The inconsistent association between elevated blood pressure and frailty in our study also concurs with the past systematic review showing the similar conflicting association between elevated blood pressure and frailty (Vetrano et al., 2018). The inconsistencies across the studies might stem from the heterogeneity in the operationalization of cardiometabolic risk factors and frailty, study design, and population. More studies are needed to confirm the association between dyslipidemia, elevated blood pressure, and frailty using uniform assessment criteria to quantify frailty.

Although this study did not aim to examine the causal mechanisms between cardiometabolic risk factors and frailty, existing literature describes the potential associations between cardiometabolic risk factors and frailty. Cardiometabolic risk factors induce inflammation even in the absence of a pathological agent. Abdominal obesity, a marker of visceral fat, instigates a state of chronic low-grade inflammation (Vandanmagsar et al., 2011) and increases the release of proinflammatory markers and chemokines such as interleukin (ILs) and TNFs (Dahlén et al., 2014; Hermsdorff et al., 2011). Similarly, hyperglycemia induces inflammatory processes characterized by increased levels of cortisol, proinflammatory cytokines, and reactive oxygen species (Keaney et al., 2003; Kir et al., 2019; Stentz et al., 2004). HDL is demonstrated to have anti-inflammatory properties, and lower HDL along with higher TC, triglycerides, and LDL appear in several chronic diseases, indicating the inflammatory role of dyslipidemia (Batuca et al., 2009; Can et al., 2015; Pietrzak et al., 2019). Moreover, hypertension can result from inflammatory changes, and it can further aggravate inflammatory changes (Idris-Khodja et al., 2014; Nosalski et al., 2017). A substantial body of evidence shows that chronic low-grade inflammation is linked to a loss of muscle mass, muscle anabolism, reduced muscle strength, and poor handgrip strength (Bano et al., 2017; Gale et al., 2013; Tuttle et al., 2020)—hallmarks of frailty. Thus, a strong body of evidence suggests that chronic inflammatory changes instigated by cardiometabolic risk factors can perpetuate frailty (Soysal et al., 2016).

Our findings suggest several research implications. First, there is a wide variation in the existing studies regarding the operationalization of frailty and cardiometabolic risk factors; thus, the use of uniform measures of frailty and cardiometabolic risk factors and consistent study design, follow-up, covariates will help in quantitative comparison and combination of findings. Although existing studies show that co-occurrence of multiple cardiometabolic risk factors is associated with a greater likelihood of frailty, the composition of co-occurring cardiometabolic factors would be more beneficial in tailoring interventions to manage and prevent frailty. Thus, identifying the subgroups of older adults with the unique combinations of cardiometabolic risk factors and their association with frailty/frailty features could enhance precision-based care. In addition, future studies should examine the associations between cardiometabolic risk factors and frailty and the role of underlying inflammatory mechanisms. A better understanding of mechanisms underlying the association between cardiometabolic risk factors and frailty can inform treatment strategies. Future studies should examine how associations between cardiometabolic risk factors and frailty vary by sociodemographic attributes such as gender, race, and ethnicity. More studies are needed to explore whether antecedent life determinants, including exposure to biopsychosocial stressors, influence the associations between cardiometabolic risk factors and frailty. Examining gender, race/ethnic stratification, and biopsychosocial stressors influencing the associations between cardiometabolic risk factors and frailty would help allocate resources for tailored/target health programs to prevent and manage frailty. In clinical settings, health care professionals need to prioritize the assessment of frailty among patients with greater cardiometabolic risk factors. Care providers should be cautious of frailty in those with cardiometabolic risk before devising a care and treatment strategy.

### Strengths and Limitations

The main strength of this systematic review was the rigorous methodologies involved in identifying and selecting the existing studies from multiple databases. This study is the first to synthesize the association between a range of potentially modifiable cardiometabolic risk factors and frailty. Future studies can use comprehensive search strategies to replicate data collection. The risk of bias of each study was evaluated using widely accepted tools. However, our findings should be interpreted in light of several limitations. The included studies had cross-sectional and longitudinal designs, restricting causal associations between cardiometabolic risk factors and frailty. The effect estimates demonstrating the associations between cardiometabolic risk factors and frailty were heterogeneous, which could stem from variation across the studies in terms of (a) clinical characteristics (participants’ age, race/ethnicity, gender, cardiometabolic risk severity, and clinical comorbidities) and (b) methodological characteristics (settings, designs; and sample size, operationalization of cardiometabolic risk factors, frailty, and covariates). The pooled estimate could not be computed due to clinical and methodological heterogeneity across the studies, limiting the generalizability of findings and comparison of results with other reviews (Lee, 2019).

## Conclusion

Our review demonstrates that hyperglycemia, abdominal obesity, and co-occurrence of multiple cardiometabolic risk factors are associated with a greater likelihood of frailty among older adults. More studies are needed to validate the association between other cardiometabolic risk factors, including dyslipidemia and elevated blood pressure, and frailty. Our review revealed considerable heterogeneity in the study population, designs, and operationalization of frailty, cardiometabolic risk factors, and covariates, limiting the quantitative synthesis of findings. Therefore, more studies are needed to examine the association between cardiometabolic risk factors and frailty using the uniform definition of frailty and cardiometabolic risk factors adjusting for potential covariates. Future studies need to identify specific combinations of cardiometabolic risk factors in older adults and their associations with frailty to inform the precision-based clinical intervention and practice. Including multiracial and ethnic groups in future studies will help understand the differential impact of cardiometabolic risk burden on frailty and help identify high-risk groups.

## Supplementary Material

igac032_suppl_Supplementary_Material
