# Supplementary material for: The Association Between Cardiometabolic Risk Factors and Frailty in Older Adults: A Systematic Review
Source: Innov Aging. 2022 May 25;6(5):igac032. doi: 10.1093/geroni/igac032 (PMC9250659; doi:10.1093/geroni/igac032)
Supplement: igac032_suppl_Supplementary_Material [file igac032_suppl_supplementary_material.docx]

| Supplementary Table 1. Search terms in PubMed, Elsevier, and Web of Science | |
| --- | --- |
| Concepts | Search terms |
| Population | Terms used:  "Frail Elderly"[Mesh] OR "Aged"[Mesh] OR “Aged, 60 and over"[Mesh] OR "Geriatrics"[mesh] OR elderly[tiab] OR aged[tiab] OR geriatric[tiab] OR geriatrics[tiab] OR elderly[tiab] OR elder[tiab] OR elders[tiab] OR "older people"[tiab] OR "older person"[tiab] OR senior[tiab] OR seniors[tiab] |
| Exposure | "Cardiovascular Diseases"[Mesh] OR "Heart Disease Risk Factors"[Mesh] OR "Cardiometabolic Risk Factors"[Mesh] OR "Obesity, Abdominal"[Mesh] OR "Blood Pressure"[Mesh] OR "Blood Glucose"[Mesh] OR "Insulin"[Mesh] OR "Lipoproteins"[Mesh] OR "Metabolic Syndrome"[Mesh] OR "Diabetes Mellitus"[Mesh] OR "Hypertension"[Mesh] OR "Obesity"[Mesh] OR cardiovascular[tiab] OR metabolic[tiab] OR cardiometabolic[tiab] OR "blood pressure"[tiab] OR "blood sugar" OR "blood glucose"[tiab] OR cholesterol[tiab] OR triglycerides[tiab] OR triglyceride[tiab] OR lipoprotein[tiab] OR lipoproteins[tiab] OR obesity[tiab] OR "heart disease"[tiab] OR diabetes[tiab] OR insulin[tiab] |
| Outcomes | "Frailty"[Mesh] OR Frail[tiab] OR frailty[tiab] OR Frailties[tiab] OR Frailness[tiab] |
| Studies | "Single-Blind Method"[Mesh] OR "Double-Blind Method"[Mesh] OR "Cohort Studies"[Mesh] OR "Follow-Up Studies"[Mesh] OR "Longitudinal Studies"[Mesh] OR "Prospective Studies"[Mesh] OR "Retrospective Studies"[Mesh] OR "Case-Control Studies"[Mesh] OR "Cross-Sectional Studies"[Mesh] OR "Controlled Before-After Studies"[Mesh] OR "Interrupted Time Series Analysis"[Mesh] OR "Cross-Over Studies"[Mesh] OR "Randomized Controlled Trials as Topic"[Mesh] OR "Meta-analysis as topic"[Mesh] OR "Evaluation studies as topic"[Mesh] OR "Randomized Controlled Trial"[pt] OR "Controlled Clinical Trial"[pt] OR "Clinical Trial"[pt] OR "Observational Study"[pt] OR "Evaluation Studies"[pt] OR "Comparative Study"[pt] OR "Meta-Analysis"[pt] OR "Systematic Review"[pt] OR systematic[subset] OR randomized[tiab] OR randomised[tiab] OR randomization[tiab] OR randomisation[tiab] OR placebo[tiab] OR randomly[tiab] OR trial[tiab] OR trials[tiab] OR groups[tiab] OR "single blind"[tiab] OR "single blinded"[tiab] OR "double blind"[tiab] OR "double blinded"[tiab] OR "evaluation study"[tiab] OR "evaluation studies"[tiab] OR "intervention study"[tiab] OR "intervention studies"[tiab] OR cohort[tiab] OR cohorts[tiab] OR longitudinal[tiab] OR longitudinally[tiab] OR prospective[tiab] OR prospectively[tiab] OR retrospective[tiab] OR retrospectively[tiab] OR follow-up[tiab] OR "follow up"[tiab] OR followup[tiab] OR case-control[tiab] OR "case control"[tiab] OR "case controlled"[tiab] OR case-controlled[tiab] OR cross-sectional[tiab] OR "cross sectional"[tiab] OR crossover[tiab] OR "cross over"[tiab] OR "comparative study"[tiab] OR "comparative studies"[tiab] OR meta-analysis[tiab] OR meta-analyses[tiab] OR "meta-analysis"[tiab] OR "meta analyses"[tiab] OR metaanalysis[tiab] OR metaanalyses[tiab] OR meta-analytic[tiab] OR "meta analytic"[tiab] OR metaanalytic[tiab] |
| Note: The results of search are shown in Appendix. | |

| Supplementary Table 2. Search results in databases─ CINAHL (via EBSCO), Embase (via Elsevier), and MEDLINE (via new PubMed). | | |
| --- | --- | --- |
| Search | Query | Results |
| 1  frailty | "Frailty"[Mesh] OR Frail[tiab] OR frailty[tiab] OR Frailties[tiab] OR Frailness[tiab] | 24,817 |
| 2 | "Cardiovascular Diseases"[Mesh] OR "Heart Disease Risk Factors"[Mesh] OR "Cardiometabolic Risk Factors"[Mesh] OR "Obesity, Abdominal"[Mesh] OR "Blood Pressure"[Mesh] OR "Blood Glucose"[Mesh] OR "Insulin"[Mesh] OR "Lipoproteins"[Mesh] OR "Metabolic Syndrome"[Mesh] OR "Diabetes Mellitus"[Mesh] OR "Hypertension"[Mesh] OR "Obesity"[Mesh] OR cardiovascular[tiab] OR metabolic[tiab] OR cardiometabolic[tiab] OR "blood pressure"[tiab] OR "blood sugar" OR "blood glucose"[tiab] OR cholesterol[tiab] OR triglycerides[tiab] OR triglyceride[tiab] OR lipoprotein[tiab] OR lipoproteins[tiab] OR obesity[tiab] OR "heart disease"[tiab] OR diabetes[tiab] OR insulin[tiab] | 4,320,907 |
| 3  elderly | "Frail Elderly"[Mesh] OR "Aged"[Mesh] OR "Aged, 80 and over"[Mesh] OR "Geriatrics"[mesh] OR elderly[tiab] OR aged[tiab] OR geriatric[tiab] OR geriatrics[tiab] OR elderly[tiab] OR elder[tiab] OR elders[tiab] OR "older people"[tiab] OR "older person"[tiab] OR senior[tiab] OR seniors[tiab] | 3,720,480 |
| 4 | 1 and 2 and 3 | 4282 |
| 5 | 4 NOT (Editorial[ptyp] OR Letter[ptyp] OR Comment[ptyp]) NOT (animals[mesh] NOT humans[mesh]) | 4091 |
| 6 | "Single-Blind Method"[Mesh] OR "Double-Blind Method"[Mesh] OR "Cohort Studies"[Mesh] OR "Follow-Up Studies"[Mesh] OR "Longitudinal Studies"[Mesh] OR "Prospective Studies"[Mesh] OR "Retrospective Studies"[Mesh] OR "Case-Control Studies"[Mesh] OR "Cross-Sectional Studies"[Mesh] OR "Controlled Before-After Studies"[Mesh] OR "Interrupted Time Series Analysis"[Mesh] OR "Cross-Over Studies"[Mesh] OR "Randomized Controlled Trials as Topic"[Mesh] OR "Meta-analysis as topic"[Mesh] OR "Evaluation studies as topic"[Mesh] OR "Randomized Controlled Trial"[pt] OR "Controlled Clinical Trial"[pt] OR "Clinical Trial"[pt] OR "Observational Study"[pt] OR "Evaluation Studies"[pt] OR "Comparative Study"[pt] OR "Meta-Analysis"[pt] OR "Systematic Review"[pt] OR systematic[subset] OR randomized[tiab] OR randomised[tiab] OR randomization[tiab] OR randomisation[tiab] OR placebo[tiab] OR randomly[tiab] OR trial[tiab] OR trials[tiab] OR groups[tiab] OR "single blind"[tiab] OR "single blinded"[tiab] OR "double blind"[tiab] OR "double blinded"[tiab] OR "evaluation study"[tiab] OR "evaluation studies"[tiab] OR "intervention study"[tiab] OR "intervention studies"[tiab] OR cohort[tiab] OR cohorts[tiab] OR longitudinal[tiab] OR longitudinally[tiab] OR prospective[tiab] OR prospectively[tiab] OR retrospective[tiab] OR retrospectively[tiab] OR follow-up[tiab] OR "follow up"[tiab] OR followup[tiab] OR case-control[tiab] OR "case control"[tiab] OR "case controlled"[tiab] OR case-controlled[tiab] OR cross-sectional[tiab] OR "cross sectional"[tiab] OR crossover[tiab] OR "cross over"[tiab] OR "comparative study"[tiab] OR "comparative studies"[tiab] OR meta-analysis[tiab] OR meta-analyses[tiab] OR "meta analysis"[tiab] OR "meta analyses"[tiab] OR metaanalysis[tiab] OR metaanalyses[tiab] OR meta-analytic[tiab] OR "meta analytic"[tiab] OR metaanalytic[tiab] | 8,465,068 |
| 7 | 5 AND 6 | 2901 |

| Supplementary Table 3. Results of search in database (including vendor/ platform): Web of Science (via Clarivate) | | |
| --- | --- | --- |
| Search | Query | Results |
| 1  frailty | TS=(frail OR frailty OR frailties OR frailness) | 37,798 |
| 2 | TS=(cardiovascular OR metabolic OR cardiometabolic OR "blood pressure" OR "blood sugar" OR "blood glucose" OR cholesterol OR triglycerides OR triglyceride OR lipoprotein OR lipoproteins OR obesity OR "heart disease" OR diabetes OR insulin) | 2,777,740 |
| 3  elderly | TS=(aged OR geriatric OR geriatrics OR elderly OR elder OR elders OR "older people'" OR "older person'" OR senior OR seniors) | 4,925,183 |
| 4 | #1 and #2 and #3 | 5,237 |
| 5 | #4 AND ALL=(randomized OR randomised OR randomization OR randomisation OR placebo OR randomly OR trial OR trials OR groups OR "single blind" OR "single blinded" OR "double blind" OR "double blinded" OR "evaluation study" OR "evaluation studies" OR "intervention study" OR "intervention studies" OR cohort OR cohorts OR longitudinal OR longitudinally OR prospective OR prospectively OR retrospective OR retrospectively OR follow-up OR "follow up" OR followup OR case-control OR "case control" OR "case controlled" OR case-controlled OR cross-sectional OR "cross sectional" OR crossover OR "cross over" OR "comparative study" OR "comparative studies" OR meta-analysis OR meta-analyses OR "meta analysis" OR "meta analyses" OR metaanalysis OR metaanalyses OR meta-analytic OR "meta analytic" OR metaanalytic) | 3,701 |
| 6 | #5 AND Articles or Review Articles (Document Types) | 3,638 |

*TS*: Term search

| Supplementary Table 4. Quality appraisal of studies using the Joana Briggs Instrument | | | | | | | |
| --- | --- | --- | --- | --- | --- | --- | --- |
| Name of article | Sample inclusion criteria | Study subjects and settings | Valid and reliable measurement of exposure | Identification of confounding factors | Adjustment for confounding factors | Valid and reliable measurement of outcomes | Appropriate statistical analysis techniques |
| Crow | Yes | Yes | Yes | Yes | Yes | Yes | Yes |
| Liao | Yes | Yes | Yes | Yes | Yes | Yes | Yes |
| Song | Yes | Yes | Yes | Yes | Yes | Yes | Yes |
| Blaun | Yes | Yes | Yes | Yes | Yes | Yes | Yes |
| Kalyani | Yes | Yes | Yes | Yes | Yes | Yes | Yes |
| Zaslavsky | Yes | Yes | Yes | Yes | Yes | Yes | Yes |
| Anker | Yes | Yes | Yes | Yes | Yes | Yes | Yes |
| Lee | Yes | Yes | Yes | No | Yes | Yes | Yes |
| Perez-Tasigchana | Yes | Yes | Yes | Yes | Yes | Yes | Yes |
| Barzilay | Yes | Yes | Yes | Yes | Yes | Yes | Yes |
| Graciani | Yes | Yes | Yes | Yes | Yes | Yes | Yes |
| Gale | Yes | Yes | Yes | Yes | Yes | Yes | Yes |
